# Greater gulf coast regional translational workforce development: Assessment and action plan

**DOI:** 10.1017/cts.2025.31

**Published:** 2025-02-18

**Authors:** Courtney D. Hunt, Richard Sucgang, Ming Guo, Glenn Sanford, Dorothy E. Lewis, Melinda Sheffield-Moore, Rebecca M. Hall

**Affiliations:** 1 Houston Methodist, Houston, TX, USA; 2 The University of Texas Medical Branch, Galveston, TX, USA; 3 University of Houston-Clear Lake, Houston, TX, USA

**Keywords:** Workforce, clinical and translational research administration, translational science, biomedical, academic medical centers

## Abstract

Converting knowledge from basic research into innovations that improve clinical care requires a specialized workforce that converts a laboratory invention into a product that can be developed and tested for clinical use. As the mandate to demonstrate more real-world impact from the national investment in research continues to grow, the demand for staff that specialize in product development and clinical trials continues to outpace supply. In this study, two academic medical institutions in the greater Houston–Galveston region termed this population the “bridge and clinical research professional” (B + CRP) workforce and assessed its turnover before and after the onset of the COVID-19 pandemic . Both institutions realized growth (1.2 vs 2.3-fold increase) in B + CRP-specific jobs from 2017 to 2022. Turnover increased 1.5–2-fold after the onset of the pandemic but unlike turnover in the larger clinical and translational research academic workforce, the instability did not resolve by 2022. These results are a baseline measurement of the instability of our regional B + CRP workforce and have informed the development of a regional alliance of universities, academic medical centers, and economic development organizations in the greater Houston–Galveston region to increase this highly specialized and skilled candidate pool.

## Introduction

**Figure 1.**
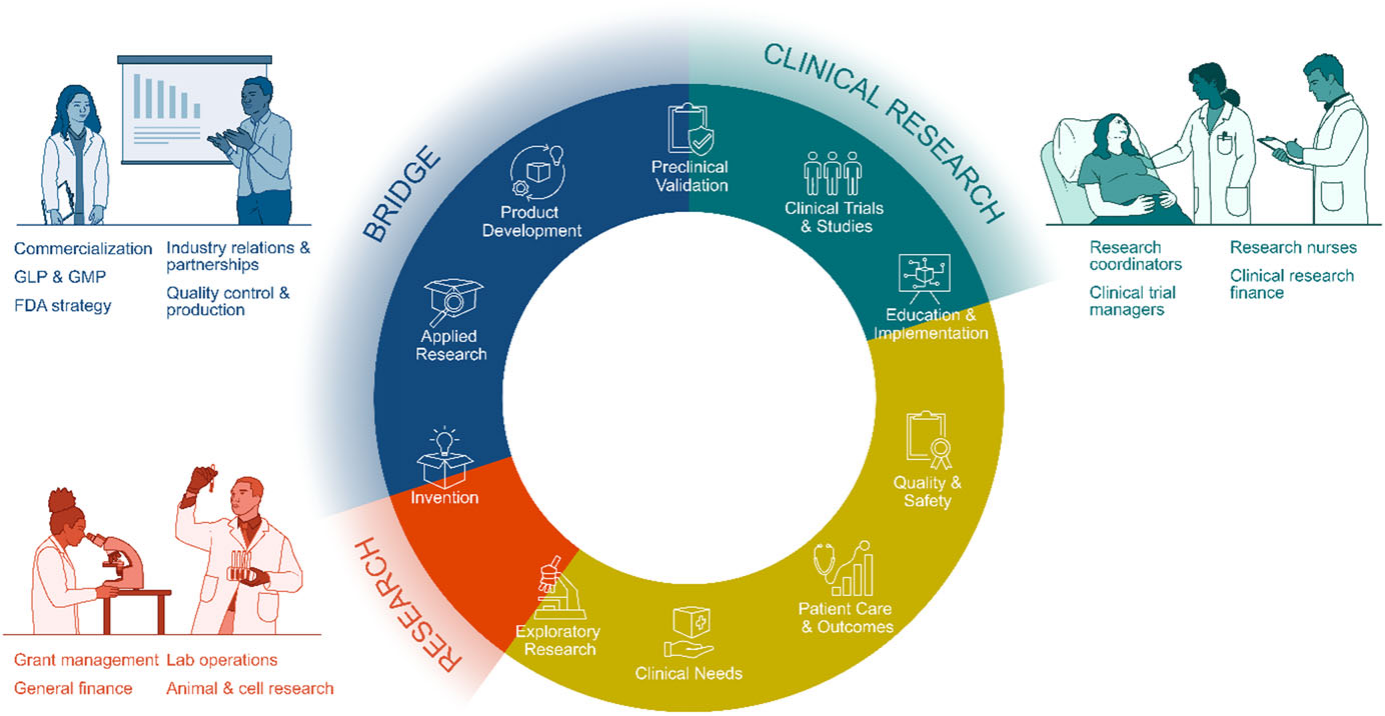
Staff (nonfaculty) positions critical to bridge basic research discoveries through clinical research into clinical use.

The National Institutes of Health (NIH) established the National Center for Advancing Translational Sciences (NCATS) in 2011 to realize the promise of basic science by increasing translation of knowledge into real-world clinical and health impact for the American people [1]. Along the spectrum of translation, basic research in the laboratory uncovers fundamental mechanisms of biology and disease, which is further elucidated to understand a disorder and discover ways to treat it. Many steps are then needed to bridge the gap or translate basic research into clinical care, including applied research to test, evaluate and refine new materials, devices, systems or methods into a final experimental product or process; design and conduct pilot studies of investigational drugs and device prototypes within an FDA complaint controlled environment; preclinical validation through FDA compliant good laboratory processes; and FDA approval for entry into clinical trials and/or clinical use (Figure 1) [2].

Successfully traversing this process requires specialized knowledge, resources, and infrastructure that is distinct from that utilized for basic laboratory research. The network of institutions in the Clinical and Translational Science Awards (CTSA) Program has focused on skills training and process improvements to support translation. In 2016, NCATS began to address the regulatory, inter and intra-institutional administrative and operational inefficiencies in clinical trials that had to be resolved to accelerate impact [3]. The CTSA mandate from NCATS is now to advance the “Science of Translation” to accelerate the administrative and operational process of leveraging new knowledge to create real-world impact in the increasingly complex research ecosystems and systems architecture.

Recommendations for continuous improvements in preparing the next generation of independent scientists (i.e., faculty or Principal Investigator [PI] roles) have been detailed in reports from think tanks such as the National Academies of Sciences, Engineering and Medicine [4], and the Biomedical Research Workforce Working Group of the Advisory Committee to the NIH Director [5]. Institutional and individual training grants have served to raise awareness as well as prepare undergraduate, graduate, and postdoctoral trainees and residents (i.e., R25, T32, Medical Research Scholars Program) for successful research careers. In addition to developing the next generation of research scientists, it is also critical to continuously prepare nonfaculty and nontrainee staff members that contribute to moving an idea from the lab to a product that can be developed, tested, and implemented in clinical use. These staff careers are essential, yet few training pathways exist and they are not well known in the workforce marketplace. The Association of Clinical Research Professionals (ACRP) supports this notion, having noted that “career paths in clinical research are not visible or accessible to the broader workforce” [6].

The NIH, through NCATS, and the National Science Foundation, through the GRANTED program [7], have more recently recognized the importance of bolstering the biomedical research-related technical, administrative, and operational workforce that are essential to the practice of team science and academic medicine. In 2019, NCATS solicited public feedback on the CTSA program. NCATS then incorporated a key program goal to develop and implement training programs for clinical research professionals including “clinical researchers, research nurses, pharmacists, administrators, coordinators, consultants, data managers, quality assurance managers, regulatory affairs managers or educators in clinical trial management” [8]. Programs such as the NIH Broadening Experience in Scientific Training (BEST) Program prepare research trainees for broader biomedical research careers [9]. Similarly, the NSF GRANTED program was launched in 2023 to build research administration capacity and diversity nationwide, with training in pre/postaward administration, technology transfer, industry relations, research integrity, compliance, and security at the national level [7].

Despite this progress, the clinical and translational research workforce deficit is expected to continue to grow. According to the Bureau of Labor Statistics, translational research-related fields are projected to have higher than average job growth between 2022 and 2032 (e.g., clinical lab technician jobs and biological technician jobs at 5% growth and medical scientists at 10% growth [10–12]). Clinical research professional job postings had an average 9.3% compound annual growth (5.33–13.47%) from 2016 to 2019, with clinical research coordinators, clinical trial managers, and clinical trial assistant positions having more than 10% compound annual growth [6]. This corresponds with a 43% increase in new clinical research studies on clinicaltrials.gov (27,786 new studies registered in 2016; 39,723 registered in 2023) [13]. This growth increased despite, or perhaps due to, the COVID-19 pandemic. Along with the greater need for clinical research professionals due to growth in clinical trials, increased job postings [14] may also reflect healthcare worker burnout due to the COVID-19 pandemic [14,15]. Many job openings are likely replacements for workers transferring to different occupations or exiting the workforce. Given the specialized knowledge and skills that are required for positions that bridge discoveries from the laboratory into products that move through the development pipeline and into clinical trials, academic medical institutions are also competing with industry to recruit and retain this workforce. Altogether, demand for clinical research staff has outpaced supply, as a SWOG Cancer Research Network Survey of oncology research professionals found that 80% of respondents cited a clinical trial personnel shortage caused by the COVID-19 pandemic [16]. This level of growth in clinical and translational research requires an influx of talent, either through new entry to the workforce or transition from other positions. Further, diversity in the clinical and translational workforce is critical, as a less diverse research staff is correlated with decreased diversity in clinical trials, exacerbating the reduced access of underserved populations to quality healthcare [17]. While national initiatives are beginning to address the nationwide workforce deficit described above, the greater Houston–Galveston region does not have a parallel regional effort despite being home to the largest academic medical center in the world.

Here, we have undertaken a baseline study of our regional clinical and translational workforce to understand challenges specific to the Houston–Galveston region to inform the design of career development that supports the sustainability of the clinical and translational research workforce. In this manuscript, we focus on a subset of the translational workforce that is critical to moving a laboratory discovery through the product development pipeline and into clinical trials, which we have termed “Bridge and Clinical Research Professionals” (B + CRP). We define Bridge professionals as non-faculty staff who move an IP-protected invention through product development and regulatory strategy up to clinical trials, and Clinical Research Professionals as nonfaculty staff who manage and operate clinical studies. In this study, two academic medical institutions (Houston Methodist and University of Texas Medical Branch [UTMB]) within the Houston–Galveston region partnered to examine our hypotheses that the B + CRP workforce at our institutions has higher turnover compared to the overall institutional workforce, and that in contrast to other job families, this turnover had not rebounded in the years following the COVID-19 pandemic.

## Materials and methods

Per Houston Methodist Research Institute Institutional Review Board, this study was determined not to be regulated as human subjects research. No identifiable data was involved.

### Institutions

The Houston Methodist system comprises an academic research institute, eight hospitals, and a clinical trial network spanning the greater Houston metropolitan area. UTMB is a public academic medical center that predominantly serves the region spanning south from the Houston metro area to Galveston Island on the gulf coast, as well as regional clinics across the state. UTMB also houses extensive graduate and medical programs, with 3850 students in 2023. Each institution serves adjacent but distinct patient populations within the Houston–Galveston region. Both institutions are also academic medical centers with robust clinical and translational activities, making these institutions ideal for comparing clinical and translational research workforce trends.

### Defining scope of population

Examples of B + CRP positions include clinical research coordinators, clinical research nurses, clinical trial management and oversight staff, regulatory affairs and strategy staff, technology transfer and commercialization staff, and FDA-compliant quality controlled Good Laboratory Practice and Good Manufacturing Practice manufacturing and production staff. Other research administration positions not specific to moving an invention through clinical trials such as education specialists, basic science laboratory staff and other discovery science roles, data analysts, statisticians, IRB/IACUC analysts, core facility operators, grant administrators, and non-GLP comparative medicine staff were considered out of scope. Faculty were also excluded.

### Houston methodist data source

Deidentified employment data for the academic enterprise was provided by institutional human resources (HR). Positions were assessed by department and job title to determine specific in-scope departments and job titles for all subsequent analysis. All job positions at Houston Methodist as of February 2023 were assessed for relevance to bridging laboratory research to clinical trials, defined as those specifically involved with research manufacturing and production, industry relations and commercialization, and clinical research.

Deidentified employee headcount and termination data from 2017 to 2022 were mapped to in-scope and out-of-scope categories. The number of filled positions in January was considered the employee headcount for that year. Terminations included employees who voluntarily terminated employment with the institution in that calendar year. Deidentified HR data included the department ID, department description, job code, and job code description as well as demographic information including race and ethnicity (Asian, Black, White, Hispanic), gender identity (male or female), and age group (Traditional, Before 1945; Baby Boomer, 1945–1964; Gen X, 1965–1979; Gen Y, 1980–1996; Gen Z, 1997+). Demographic data was collected at employee hiring by employees self-selecting from a structured list of terms determined by the HR department, with the option to reselect as needed throughout employment. *UTMB Data Source:* The UTMB workforce is divided into academic, clinical, or institutional support enterprises; this study included positions within the academic enterprise. Any clinical positions within the academic enterprise contribute to both teaching and education. UTMB job titles are currently structured to be used broadly for both basic and translational research positions. To determine comparable B + CRP jobs in the academic enterprise, positions listed on UTMB institutional review board-approved research protocols within the calendar year, excluding faculty, were included. Additionally, employees working in UTMB’s Sealy Institute for Drug Discovery, Sealy Institute for Vaccine Sciences clinical trials employees, and in technology transfer were included. B + CRP positions were mapped to employment and demographic data and provided deidentified to the research team. Demographic data was collected at employee hiring by employees self-selecting from a structured list of terms determined by the HR department, with the option to reselect as needed throughout employment.

### Statistical analysis

Headcount and turnover were analyzed by year for overall and B + CRP positions. Employee headcount was categorized by HR job family at each institution. Turnover was defined as the number of terminations in the year/active headcount during the year.

To assess whether turnover significantly differed between B + CRP and overall categories, chi-square tests were performed comparing the expected against the observed headcounts and terminations from 2017 to 2022 by year. Data prior to 2020 were defined as the pre-COVID-19 pandemic period. The same approach examined subpopulations defined by gender, racial and ethnic grouping, and age groups, in case these factors could be linked to changes in turnover.

## Results

**Figure 2.**
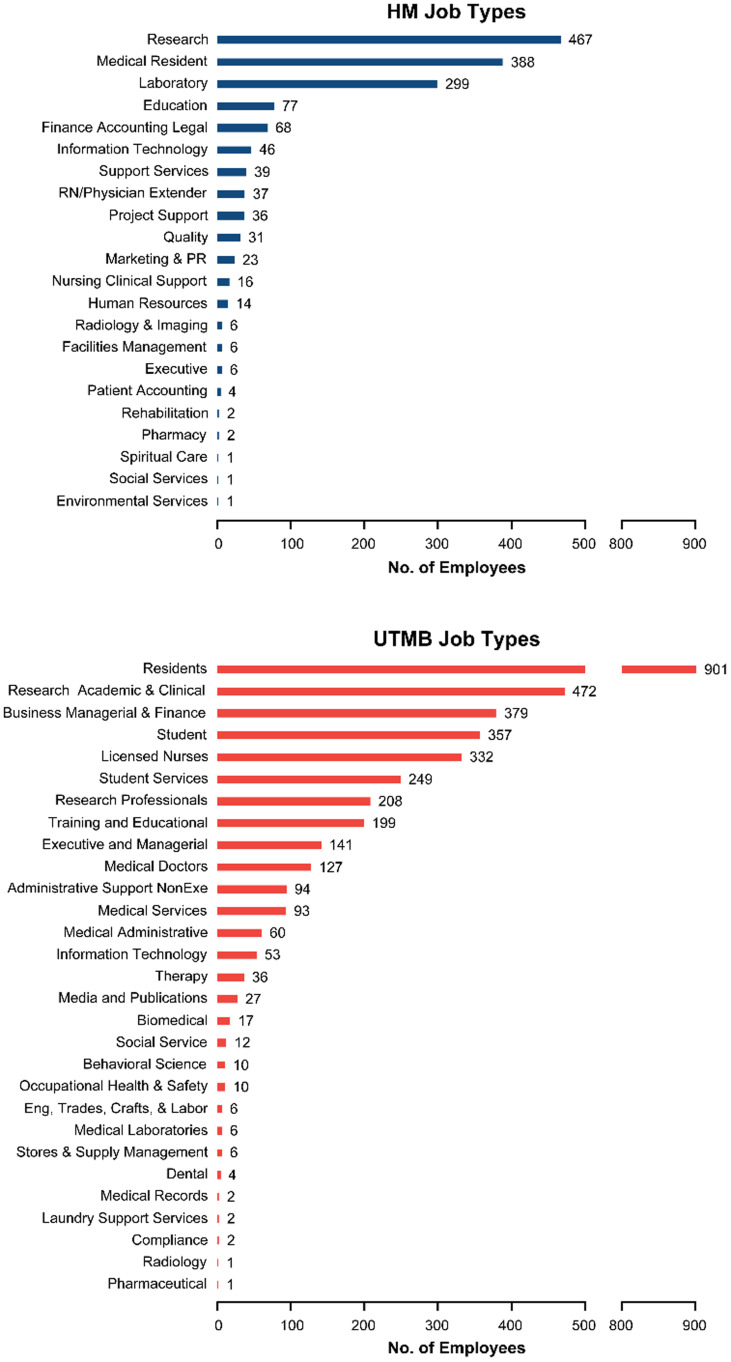
Characterization of academic workforce at Houston Methodist and UTMB. Headcounts of academic enterprise employees in 2023 were categorized by HR job families at Houston Methodist and UTMB. The academic workforce had similar job families at both institutions, though larger at UTMB (overall UTMB 2023 headcount = 3807, compared to Houston Methodist 1570).

Given the broad, nonstandardized terminology for describing the clinical and translational research workforce, we compared the structural makeup of the academic enterprise of the two academic medical centers (Figure 2). UTMB has an overall larger headcount than Houston Methodist (3807 UTMB versus 1570 Houston Methodist), but the academic workforce at both institutions contained similar job families in support of the academic mission (as distinct from corporate operations and purely clinical services, insofar as such functions could be separated in such systems). The overall similarities of the academic enterprises allowed for further characterization and comparison of workforce trends between institutions.

**Figure 3.**
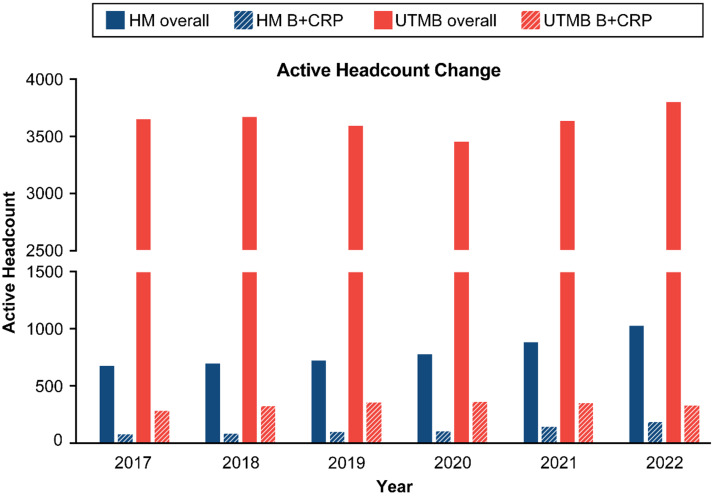
Overall academic and B + CRP workforce headcount at Houston Methodist and UTMB from 2017 to 2022. B + CRP versus overall academic workforce headcount was compared for Houston Methodist and UTMB from 2017 to 2022 to determine if B + CRP positions experienced different employment trends during the COVID-19 pandemic. Houston Methodist overall and B + CRP headcounts experienced 1.5-fold (overall, blue solid) and 2.3–fold (blue dashed, B + CRP) growth, respectively, from 2017 to 2022. UTMB B + CRP headcount experienced 17% increase (orange dashed bars), compared to 4% increase in overall academic workforce (solid orange).

The change in overall and B + CRP headcount was determined for each institution (Figure 3). Houston Methodist overall academic workforce experienced steady growth from 2017 to 2022, with overall headcount increasing 1.5-fold from 679 in 2017 to 1032 in 2022. Houston Methodist B + CRP workforce also experienced a 2.3-fold increase from 2017 to 2022 (84 in 2017 to 191 in 2022). The Houston Methodist B + CRP workforce represented 12.4% (2017) to 18.5% (2022) of the Houston Methodist academic enterprise, averaging 13.0% prepandemic (2017–2019) vs 16.6% during pandemic (2020–2022).

The UTMB overall workforce remained stable, with an overall headcount of 3654 in 2017 increasing to 3807 in 2022, whereas UTMB B + CRP workforce experienced a 1.2-fold increase from 2017 to 2022 (289 in 2017 vs 338 in 2022). UTMB B + CRP workforce represented 7.9% (2017) to 8.9% (2022) of the academic enterprise workforce (pre-pandemic average: 9% vs during-pandemic average: 9.7%).

**Figure 4.**
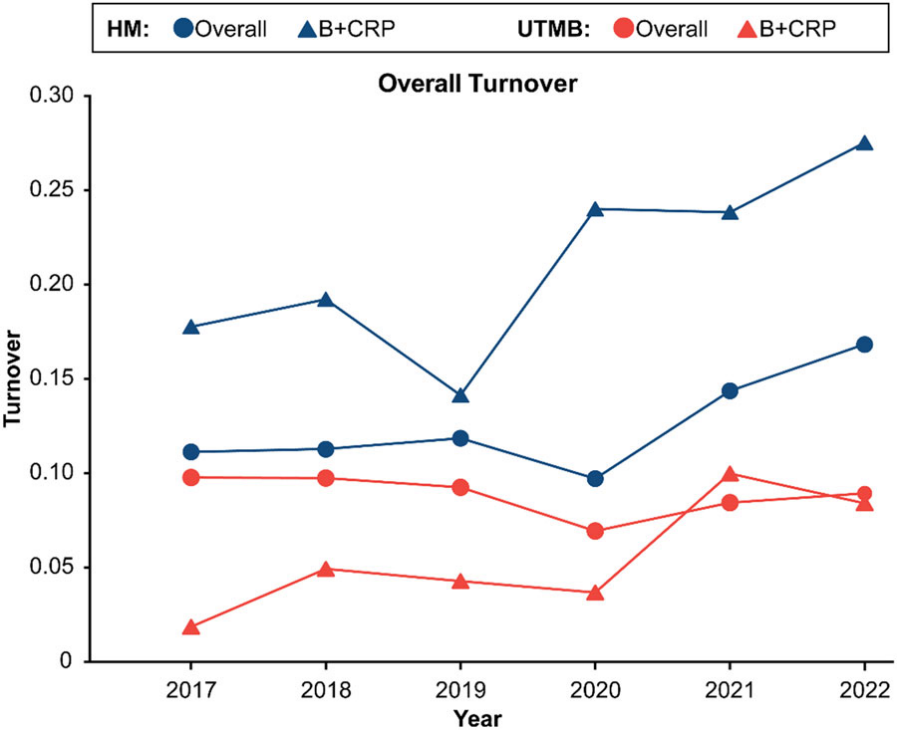
B+CRP workforce turnover increases during the COVID-19 pandemic at two academic medical centers. annualized turnover was calculated for years 2017–2022 for overall (circles) and B + CRP (triangles) positions at Houston Methodist (blue) and UTMB (orange). B + CRP positions at Houston Methodist had significantly higher turnover than overall academic workforce prepandemic, and this effect increased through the pandemic. UTMB B + CRP positions had significantly lower turnover prepandemic but reached overall-workforce turnover levels through the COVID-19 pandemic. *P* < 0.05 by Chi square test per year.

To test the hypothesis that the B + CRP workforce at our institutions experienced higher turnover compared to the overall academic workforce, turnover rate for overall academic and B + CRP workforces were compared at each institution (Figure 4). Prepandemic Houston Methodist B + CRP workforce turnover was statistically higher than overall academic turnover, and this turnover gap increased with the pandemic (17.7% B + CRP vs 11.1% overall annualized turnover in 2017; 27.4% in-scope vs 16.8% overall turnover in 2022; *p* < 0.05). Further, Houston Methodist B + CRP positions saw a 1.5-fold increase in turnover through the pandemic (16.9% average turnover 2017–2019 vs 25.0% average turnover 2020–2022). UTMB B + CRP prepandemic turnover was significantly lower than overall (1.7% B + CRP vs 9.8% overall annualized turnover in 2017, *p* < 0.05) but not during pandemic. UTMB B + CRP turnover also experienced a 2-fold increase during the pandemic, compared to prepandemic levels (3.6% average turnover 2017–2019 vs 7.2% average turnover 2020–2022).

Analysis of demographic factors (age, gender, race/ethnicity) was not significantly different for B + CRP vs overall academic turnover (Supplementary Material, Figures S1–S3).

## Discussion

A team comprised of faculty and nonfaculty professionals is required to move an idea from basic, preclinical, and clinical research through clinical implementation. In this study, we examined workforce trends for nonfaculty, B + CRP-specific workforce relative to the overall academic population at our institutions before and during the COVID-19 pandemic, ultimately to inform a model that can sustain the growing clinical and translational science workforce in our region.

One challenge we recognized as we gathered this workforce data is the inconsistency of job titles, job descriptions, and required experience between institutions, as attested elsewhere [18–20]. The HR structures at Houston Methodist and UTMB each had different job families and job titles. Job families within the academic enterprise were similar enough for this study’s comparisons (Figure 2) but diverged from there. Whereas Houston Methodist had more granular job titles and departments for translational research responsibilities, UTMB job titles were more general and had to be combined with data from IRB protocols to ensure alignment of job responsibilities and positions with Houston Methodist. Careful mapping of job positions was required to generate comparable workforce data. Our experience and those of others [18–20] indicate a need to develop consistent job titles and descriptions for the clinical and translational research workforce nationwide to provide educational and career pathways, skills development, innovation in the field, and professional growth opportunities. More consistent job positions would also facilitate future analysis and monitoring of workforce trends and evaluation of interventions. Consistent with this, the ACRP recently submitted a request to the U.S. Bureau of Labor Statistics to establish clinical researchers as a detailed occupation code within the Healthcare Practitioners and Technical Occupations job family [21].

Our findings show that the numbers of B + CRP staff employed by UTMB and Houston Methodist have increased during the last 5 years, largely during the COVID-19 pandemic. This is consistent with the increased staff needed to support steady growth in clinical trials at our institutions, as well as the national increase in clinical trials between 2017 and 2022 [13]. This outcome is also comparable to other reports indicating that the demand for clinical research workers has increased related to the pandemic [6].

Our analysis showed that the two institutions had different prepandemic B + CRP turnover trends, with B + CRP turnover higher than the overall academic workforce at Houston Methodist but lower than overall academic workforce at UTMB. It is possible that Houston Methodist B + CRP turnover was higher due to its central location in Houston, specifically within the Texas Medical Center with more competitors in proximity, compared to UTMB, which is the predominant academic medical center in the Galveston region. Both institutions experienced increased B + CRP position turnover compared to the overall academic workforce in the pandemic period. Given the increase in overall and translational headcount during this time, this is likely not due to overall reduction in United States workforce that other sectors faced with the advent of the pandemic. Given the density of clinical and translational institutions in the greater Houston–Galveston region, especially for Houston Methodist, as well as the growing potential to conduct many of these support positions remotely, higher turnover in translational positions may reflect employee movement for increased salaries, flexibility of work schedules, professional development opportunities, or job responsibilities, as experienced nationwide during the COVID-19 pandemic [22,23]. An analysis of more than 9 million employee records from 4000 global companies found that resignation rates were the highest in the technology and health care industries as the pandemic spread globally, consistent with our increased turnover data for B + CRP positions [24]. In contrast with reports that the number of women employed in research was more impacted by the pandemic and exit from workforce [25], our data did not show a significant difference in turnover based on gender, perhaps due to the sample size. Although outside the scope of this study, specialized on-boarding and retention efforts have been shown to improve employee turnover [19].

The Bay Area Houston Economic Partnership lists “healthcare and life sciences” as one of the five major drivers of the economy in the greater Houston–Galveston region [26], indicating the importance of a sustainable workforce in this sector. The increased turnover and slow recovery of the B + CRP workforce that we found suggest that action is needed to bring new workers into this niche. Although STEM careers such as nurses and physicians are high profile, other careers such as clinical coordinator, research nurse, clinical research administrator, and regulatory affairs manager are much less familiar to those working outside clinical and translational science. These careers also do not have dedicated schools; new degree programs and internship programs are broad, not yet well-known, and are more common outside the Houston–Galveston region. To ensure a sustainable supply of candidates, we believe that this region needs to develop pathways for B + CRP careers that work in this ecosystem.

One possible approach is to leverage a new “Greater Gulf Coast” regional alliance (that includes Houston Methodist and UTMB) which was formed in 2023 with the mission to build a sustainable biomedical ecosystem that brings new treatments to patients more efficiently. This alliance meets monthly and is very active in building “connectors” between undergraduate universities, academic medical centers, and regional economic partnership organizations to find ways to sustain the clinical translational science workforce and ecosystem in our region (Figure S4). The benefit of leveraging this type of alliance is the immediate multi-institutional reach for new programs which would source workforce candidates from the many local private and state-funded undergraduate institutions, some of which are designated as minority-serving institutions (University of Houston-Clear Lake and Texas Southern University). Undergraduate trainees from a wide variety of backgrounds and transferable skills in areas such as business, finance, administration, law, education, communications, and social sciences could fulfill the region’s clinical translational workforce needs. The alliance also includes connections to programs for the underemployed and unemployed through regional economic development organizations like the Greater Houston Partnership and the Bay Area Houston Advanced Technology Consortium.

### Study limitations

Each economic area and ecosystem is unique; thus, our proposed alliance model is likely to be adaptable rather than directly generalizable. Others nationwide should consider how our observations and strategies suit their local ecosystems. Resources and organizations specific to each region and at the national level such as the University-Industry Demonstration Partnership (UIDP) exist to assist industry–academic partnerships, including workforce development [27]. Although the overall similarities of our academic enterprises allowed for the comparison of workforce trends between institutions, differences in HR strategies prevented us from comparing employment data at the job title level. This reflects broader heterogeneity in job titles, descriptions, and required experience that may hinder the recruitment and retention of clinical research professionals as they move between institutions throughout their careers [28]. Small numbers within subgroups also may have masked more insight into trends in employment differences based on demographic factors. Finally, this study focused on a specialized subset of the research workforce; while outside the scope of this study, additional critical roles that support research, such as grant administrators, scientific writers, biostatisticians, and research compliance can also be considered less visible careers warranting investment in workforce development programs.

In the future, we plan to follow up with periodic assessments of the B + CRP workforce to track needs and correlations with economic changes and recovery programs implemented in our region. We will also continue to evaluate and disseminate the impact of workforce development programs that follow from this needs assessment and support the clinical and translational ecosystem of the Houston––Galveston region.

## Supporting information

Hunt et al. supplementary material Hunt et al. supplementary material

## Supplementary material

The supplementary material for this article can be found at https://doi.org/10.1017/cts.2025.31.
