# Supplementary material for: Greater gulf coast regional translational workforce development: Assessment and action plan
Source: J Clin Transl Sci. 2025 Feb 18;9(1):e62. doi: 10.1017/cts.2025.31 (PMC11975780; doi:10.1017/cts.2025.31)
Supplement: Hunt et al. supplementary material [file S2059866125000317sup001.docx]

**Title:** Greater Gulf Coast Regional Translational Workforce Development: Assessment and Action Plan

**Author Names**

Courtney D Hunt

Richard Sucgang

Ming Guo

Glenn Sanford

Dorothy E Lewis

Melinda Sheffield-Moore

Rebecca M Hall^*^

**Supplementary Materials:**


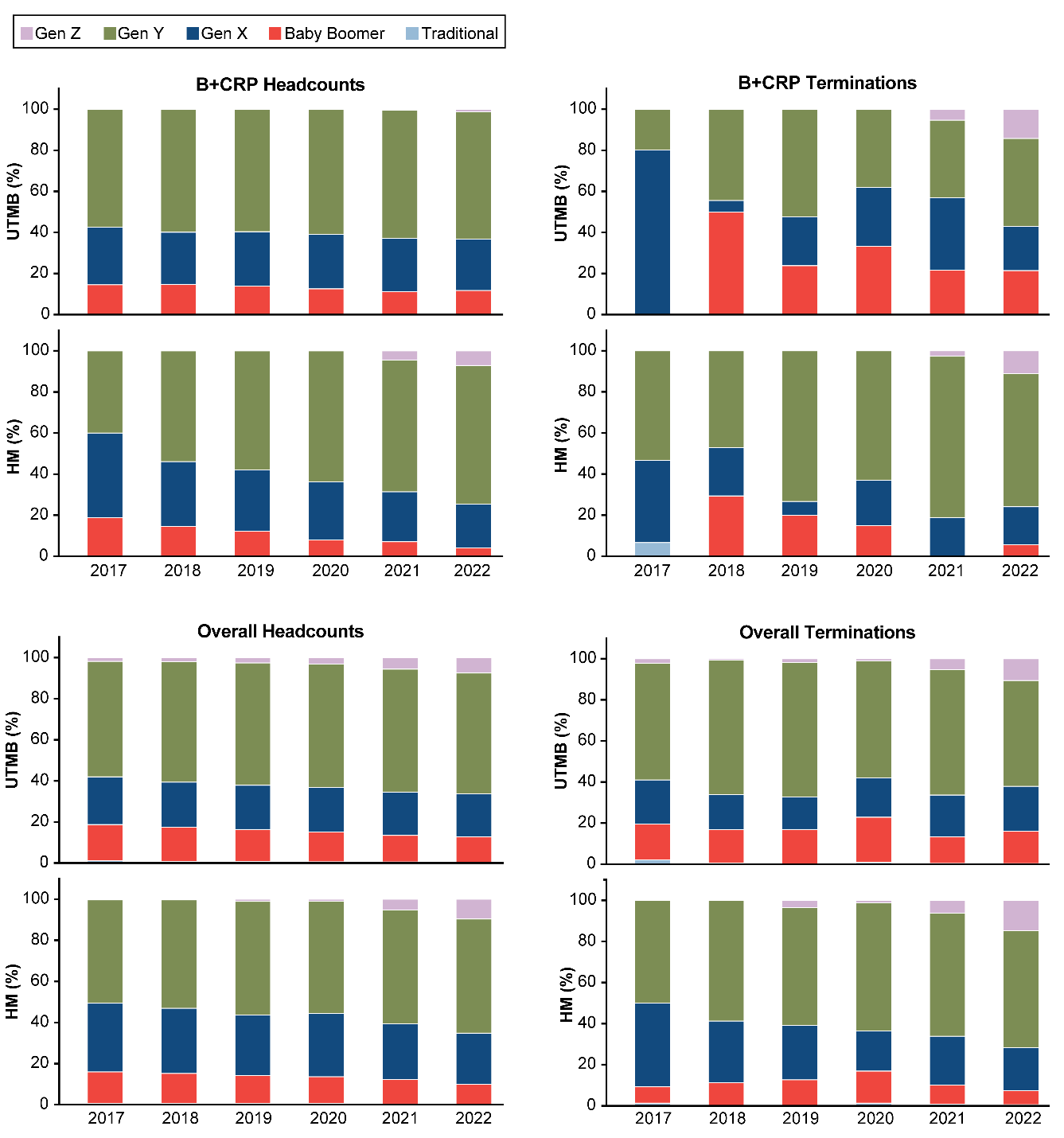


**Figure S1.** Age does not explain differences in translation-specific headcount and turnover during the COVID-19 pandemic. B+CRP and overall academic workforce headcounts (left panel) and turnover (right panel) were assessed by age group. Gen Z (pink) represents those born 1997+; Gen Y (green) represents those born 1980-1996); Gen X (dark blue) represents those born 1965-1979; Baby Boomer (red) represents those born 1945-1964; Traditional (light blue) represents those born before 1945. Changes in the aging composition of the workforce did not significantly correlate with differences in B+CRP vs overall turnover during the transition into the COVID-19 pandemic.


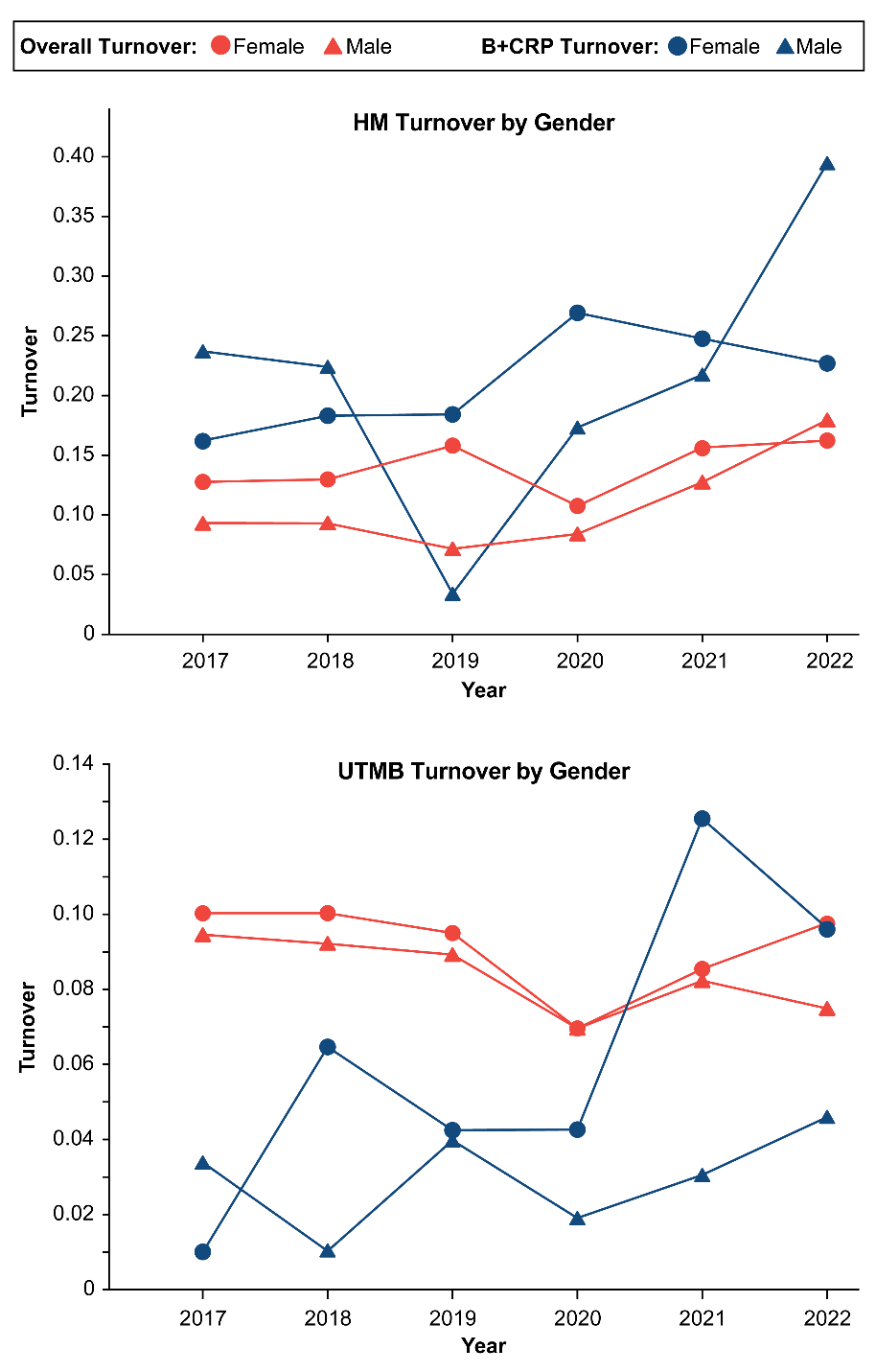


**Figure S2.** Differences in translation-specific headcount and turnover during the COVID-19 pandemic did not segregate by gender identity. B+CRP and overall turnover were assessed by self-reported gender identity at Houston Methodist (Top panel) and UTMB (Bottom panel). Differences in B+CRP (blue lines) vs. overall (red lines) turnover during the COVID-19 pandemic was not significantly explained by gender differences. However, there are institutional differences in gender-specific turnover, with B+CRP females seeing a higher turnover at UTMB during the pandemic (bottom panel, blue circles), whereas B+CRP males saw higher turnover at Houston Methodist during the pandemic (top panel, blue triangles).


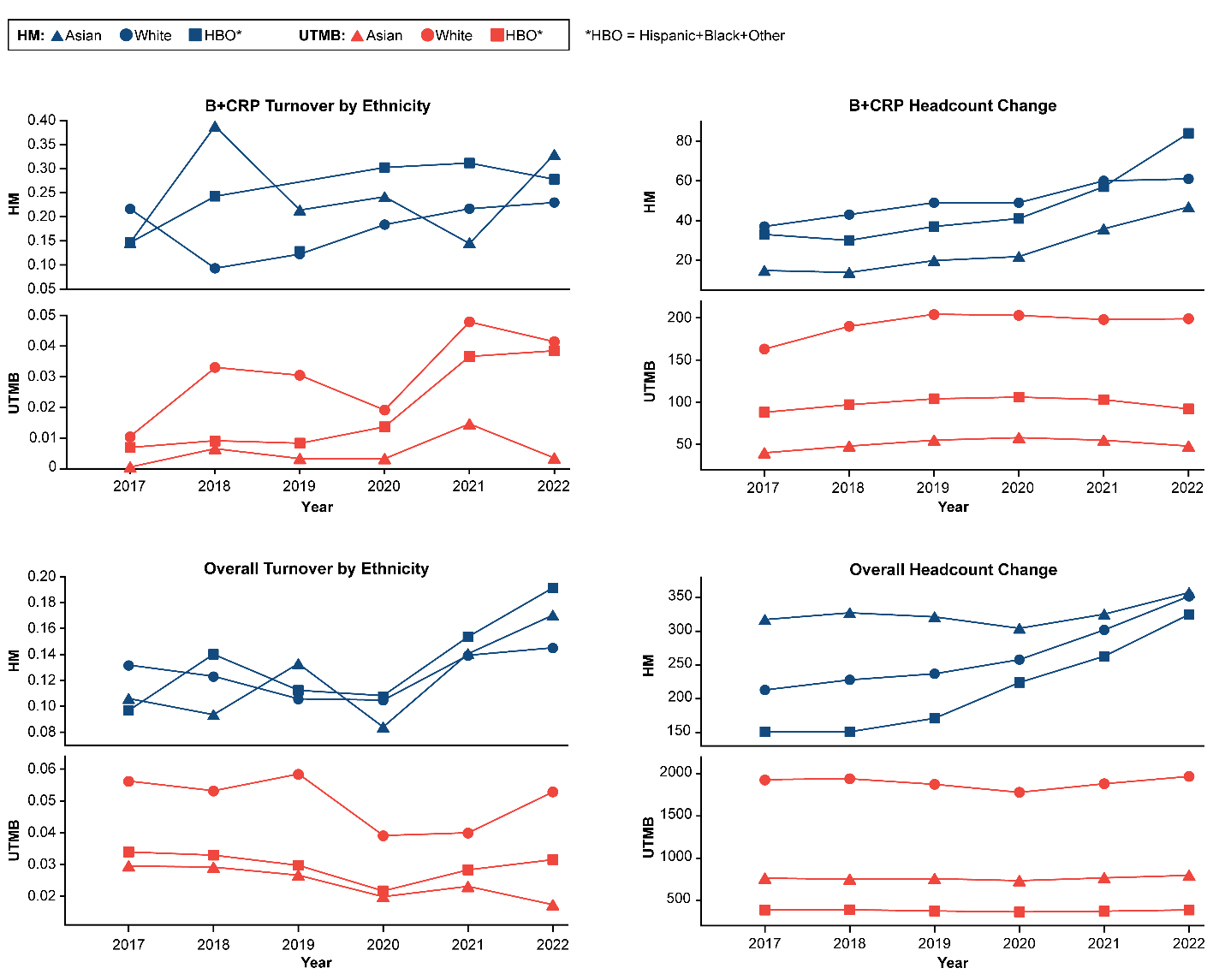


**Figure S3.** B+CRP and overall turnover and headcount by race/ethnicity. In general, within each institution, each of the defined racial/ethnic groups experienced similar trajectories of B+CRP-specific and overall workforce turnover over the period surveyed (the only exception is Asian population at Houston Methodist undergoing dramatic variation in translation-specific workforce turnover). Each institution demonstrates different general trajectories of racial/ethnic representation over time, with UTMB being relatively stable while the smaller Houston Methodist changed in proportion over time.


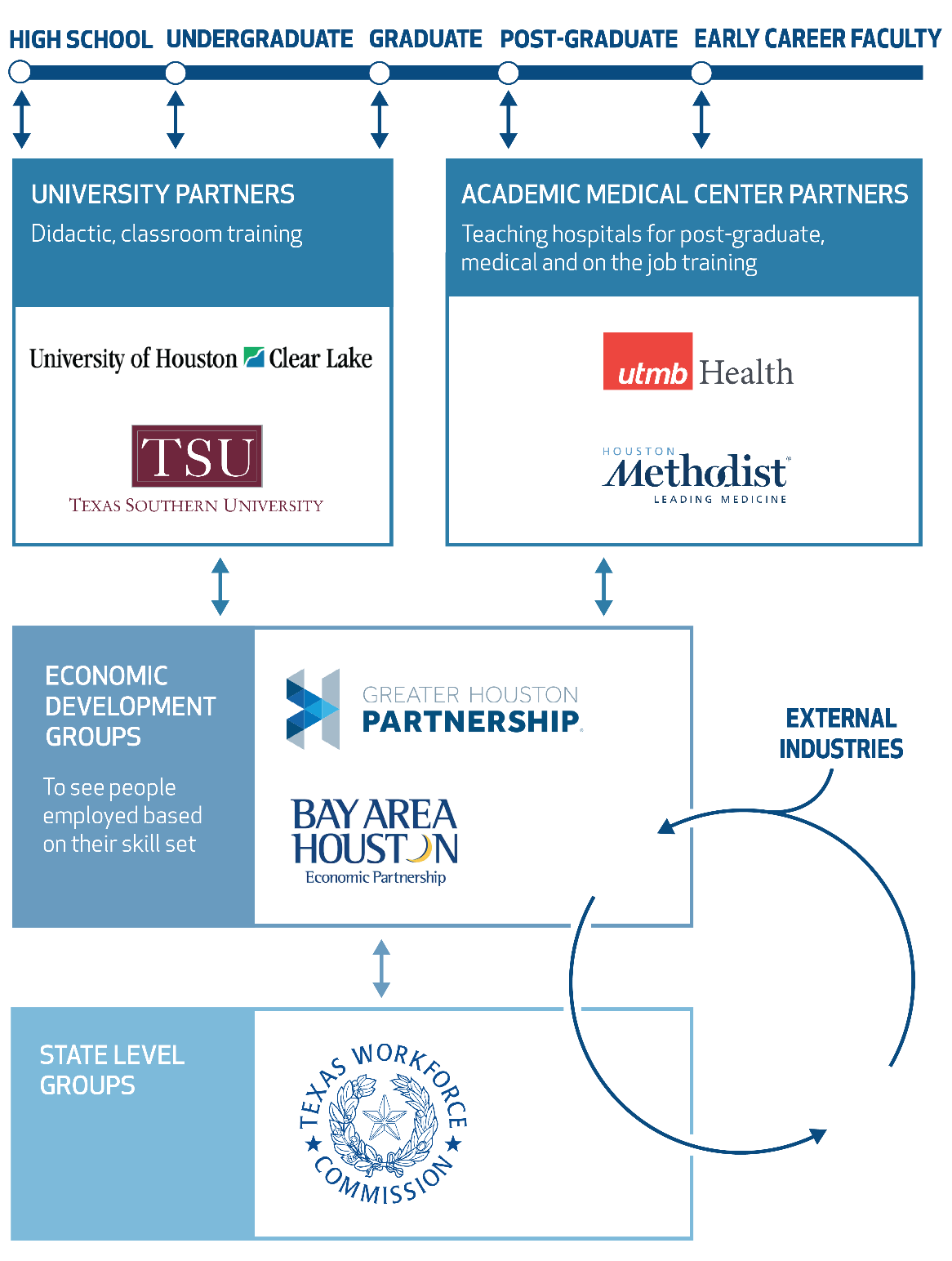


**Figure S4.** Strategic alliance to bolster the clinical and translational workforce. “Connectors” model between undergraduate universities, academic medical centers, and regional economic partnership organizations to increase awareness of critical career opportunities to best sustain the clinical translational science effort in our region.
